# Differences in metacognition between multiple sclerosis phenotypes: cognitive impairment and fatigue are key factors

**DOI:** 10.3389/fpsyg.2023.1163112

**Published:** 2023-08-23

**Authors:** Clàudia Coll-Martinez, Judit Salavedra-Pont, Maria Buxó, Ester Quintana, Ana Quiroga-Varela, René Robles-Cedeño, Marc Puig, Gary Álvarez-Bravo, Lluís Ramió-Torrentà, Jordi Gich

**Affiliations:** ^1^Girona Neuroimmumology and Multiple Sclerosis Unit, Neurology Department, Dr. Josep Trueta University Hospital and Santa Caterina Hospital, Girona, Spain; ^2^Neurodegeneration and Neuroinflammation Research Group, Girona Biomedical Research Institute (IDIBGI), Salt, Spain; ^3^Redes de Investigación Cooperativa Orientada a Resultados en Salud (RICORS), Red de Enfermedades inflamatorias (RD21/0002/0063), Instituto de Salud Carlos III, Madrid, Spain; ^4^Statistical Unit, Girona Biomedical Research Institute (IDIBGI), Salt, Spain; ^5^Medical Sciences Department, University of Girona, Girona, Spain

**Keywords:** cognition, metacognitive knowledge, deficit awareness, cognitive impairment, multiple sclerosis

## Abstract

**Background:**

Cognitive impairment is present in 40–65% of patients with multiple sclerosis (pwMS). Objectively measured cognitive performance often does not match patients' subjective perception of their own performance.

**Objective:**

We aimed to compare cognitive performance and subjective perception of cognitive deficits between pwMS and healthy controls (HCs), as well as the accuracy of subjective perception.

**Methods:**

In total, 54 HC and 112 pwMS (relapsing–remitting, RRMS, and progressive PMS) underwent neuropsychological evaluation and completed perceived deficit, fatigue, and anxiety–depression scales. Participants were classified according to their consistency between subjective self-evaluation of cognitive abilities and objective cognitive performance to assess accuracy. Regression models were used to compare cognitive performance between groups and explore factors explaining inaccuracy in the estimation of cognitive performance.

**Results:**

PMS showed greater and more widespread cognitive differences with HC than RRMS. No differences were found between pwMS and HC in the perception of deficit. PMS had higher ratios of overestimators. In explaining inaccuracy, fatigue and cognitive preservation were found to be risk factors for underestimation, whereas physical disability and cognitive impairment were risk factors for overestimation.

**Conclusion:**

PwMS have metacognitive knowledge impairments. This study provides new information about metacognition, data on the prevalence of impairments over a relatively large sample of PwMS, and new insights into factors explaining it. Anosognosia, related to cognitive impairment, may be present in pwMS. Fatigue is a key factor in underestimating cognition.

## Introduction

Multiple sclerosis (MS) is a chronic inflammatory demyelinating and neurodegenerative disease of the central nervous system (CNS). It represents the most common cause of non-traumatic neurological disability in young adults (Grzegorski and Losy, 2017). It is a highly heterogeneous disease with a great variety of symptoms including motor and sensitivity impairments such as visual problems, as well as mood disorders, fatigue, and cognitive impairment. Different phenotypes along the MS clinical course are relapsing–remitting (RR), secondary progressive (SP), and primary progressive (PP) (Lublin et al., 2014). RRMS is the most frequent phenotype, and it is characterised by the presence of clinical relapses and radiological activity, which usually recovers totally or partially spontaneously or is treated with corticosteroids depending on its severity. A high number of patients convert into SPMS over the disease evolution, usually in ~20 years, presenting gradually worsening (progression) and fewer relapses, with or without radiological activity. PPMS is less frequent (~20%) but more aggressive with the presence of progression since the beginning and without initial relapses (Klineova and Lublin, 2018).

Cognitive impairment (CI) is present in between 40 and 65% of patients with multiple sclerosis (pwMS) (Amato et al., 2001; Ruano et al., 2017), it may appear at the beginning of the disease (Campbell et al., 2017) and worsens over time (Ruano et al., 2017). Its prevalence changes across multiple sclerosis (MS) phenotypes and is more frequent in SPMS and PPMS (progressive MS-PMS) (Ruano et al., 2017). CI commonly affects processing speed, attention, executive function, and memory (Grzegorski and Losy, 2017; Ruano et al., 2017; Sumowski et al., 2018). Different cognitive phenotypes have recently been described within relapsing–remitting MS (RRMS) patients (Leavitt et al., 2018; Slavkovic et al., 2019) and within different MS subtypes, although the results are in line with earlier reports (Ruano et al., 2017): PMS have more pronounced and widespread CI than RRMS, suggesting that CI progresses along with the disease (De Meo et al., 2021; Podda et al., 2021). For instance, De Meo et al. (2021) proposed a classification of cognitive functions using latent profile analysis (LPA) and MRI data. They identified five cognitive phenotypes: “preserved cognition,” characterised by preserved performance; “mild-verbal memory/semantic fluency,” showing mildly decreased performance in verbal learning and memory; “mild-multidomain,” with mild impairment across multiple domains and cortical atrophy; “severe-executive/attention,” exhibiting severe impairment in attention and executive functions, associated with higher fatigue and white matter lesion load; and “severe-multidomain,” representing severe cognitive impairment. Severe cognitive phenotypes prevailed in patients with PMS, while mild impairments were more frequent in RRMS.

CI has a high impact on daily activities (Goverover et al., 2016; Campbell et al., 2017) and thus on quality of life and affectivity (Campbell et al., 2017; Slavkovic et al., 2019).

Objectively measured cognitive performance often fails to match patients' subjective perceptions of their performance. This might be due to factors such as the discrepancy between laboratory tasks and everyday life cognitive demands as well as premorbid abilities, given that they can mask cognitive decline (Sumowski et al., 2018) and mood disturbances that can affect cognitive performance (Feinstein, 2006; Chen and Goverover, 2021). Deficit awareness issues may also be present in pwMS. A growing interest in metacognition over the last 5–10 years is probably an acknowledgement of its impact on rehabilitation, compensation strategies, and disease management (Mazancieux et al., 2019), especially given new healthcare paradigms based on patient engagement and empowerment. However, evidence regarding perceived cognitive deficit in pwMS is controversial.

In terms of conceptualising and characterising metacognition or deficit perception (or awareness), there is not only a lack of consensus but also a notable variety in the terms that are used for the same or similar concepts across different studies. While it is beyond the purpose of this study to undertake a full discussion of this latter issue, we will provide some definitions for reasons of comprehension. The term “anosognosia” refers to the lack of awareness of a neurological deficit (classically hemiplegia, as described by Babinski in 1914). The term is now widely used in neuropsychology and psychiatry (Mazancieux et al., 2019); other terms describing anosognosia (e.g., deficit awareness or impaired awareness of deficits) are used indistinctly. “Metacognition” was first defined as “cognition about cognition” by Flavell in 1979 in the field of child development studies. It is based on healthy populations; thus, it refers to the normal ability to evaluate or monitor cognition. It can be divided into metacognitive knowledge (which is the global assessment of cognitive skills and beliefs about one's own functioning) and metacognitive experiences (which is online awareness or assessment of one's own performance during a task).

In summary, even though the two constructs come from different theoretical frameworks and have been historically studied separately, they are highly related since preserved metacognition is necessary to be aware of malfunction in one's own cognition (i.e., not to present anosognosia). Following these definitions, we make the assumption that preserved metacognition results in accurate assessments of one's own cognitive abilities and performances. On the other hand, metacognitive impairments are assumed to result in anosognosia or deficit awareness impairments. However, the situation may also arise that patients may also believe that they have impaired cognitive abilities when in fact they do not.

With regard to the available data on how pwMS perceive their own cognitive function (i.e., metacognitive knowledge), some authors have found objective (neuropsychological assessments) and subjective measures (questionnaires such as the Perceived Deficit Questionnaire, PDQ) to correlate (Kujala et al., 1996; Hoogervorst et al., 2001; Krch et al., 2011). PwMS have been found to report more subjective deficits than healthy controls (HCs), which considering that they performed worse on cognitive tests, could indicate an awareness of deficit (Matotek et al., 2001; Basso et al., 2008). Nevertheless, most studies report null or weak correlations between objective and subjective measures of cognitive performance and, conversely, moderate or strong correlations between subjective measures and affective variables such as depressive symptoms (Lovera et al., 2006; Julian et al., 2007; Kinsinger et al., 2010; Hanssen et al., 2014; Strober et al., 2016; Henneghan et al., 2017; McNicholas et al., 2021). Fewer studies have explored metacognition in terms of consistency, either between self-evaluation and informant evaluation or between subjective and objective evaluation. Nonetheless, it seems that metacognitive knowledge may be impaired in pwMS, especially when compared with HC (Goverover et al., 2014; Mazancieux et al., 2019; Chen and Goverover, 2021; Feinstein et al., 2021). However, it is not clear which factors explain metacognitive impairments and to what extent. Associations with cognitive impairment intensity (Goverover et al., 2005; Sherman et al., 2008; van der Hiele et al., 2012; Rosti-Otajärvi et al., 2014), educational level (Smith and Arnett, 2010), and variables such as mood or fatigue (Kinsinger et al., 2010; van der Hiele et al., 2012) have been reported. Different relationships with these variables have been found depending on the type of metacognitive impairment. Carone et al. (2005) reported that overestimation is associated with worse cognitive performance and less depression, whereas underestimation is correlated with higher levels of depression. All these findings together may suggest complex relationships between the factors, resulting in different outcomes for different patients. The literature also fails to give a clear picture regarding the prevalence of awareness or unawareness of cognitive deficits. Underestimation of cognitive performance ranges from 16 to 65%, accuracy from 33 to 69%, and overestimation from 2 to 24% between studies (Carone et al., 2005; Kinsinger et al., 2010; Smith and Arnett, 2010; van der Hiele et al., 2012).

Given that different MS phenotypes have different profiles of cognitive disturbances, it is interesting to investigate awareness of cognitive deficits across MS phenotypes. The results of the few authors that have studied perceived deficit in pwMS across different MS phenotypes (Sherman et al., 2008; Rosti-Otajärvi et al., 2014) suggest clear differences among them.

This study aims to investigate the perception of cognitive deficit among different MS phenotypes, comparing perceived deficit in relation to objective cognitive performance, and in terms of consistency, between RRMS, PMS, and HC. We also explore factors that may explain impaired awareness.

## Materials and methods

### Participants

This study is a cross-sectional sub-study of the ConnectiMS project which was approved by the Ethics Committee of the Dr. Josep Trueta University Hospital (code: 8014) and carried out at the Girona Neuroimmunology and Multiple Sclerosis Unit (Catalonia) from 2014 to 2018. All participants signed a written informed consent form before inclusion, and none received any financial compensation for their participation. In total, 112 patients and 54 HC were included. Patients were classified into two groups corresponding to their MS phenotype, according to the definition by Lublin et al. (2014): RRMS (*n* = 65) and PMS (SPMS and PPMS, *n* = 47). Exclusion criteria were illiteracy, having neurological alterations other than MS, history of traumatic brain injury, psychiatric disorder, drug or alcohol abuse, and corticosteroid use 2 months prior to the cognitive assessment. Standardised protocols, forms, and databases were used for data collection to minimise sources of bias.

### Procedures

All participants underwent neuropsychological assessment and self-administered the Perceived Deficit Questionnaire (PDQ) (Fischer et al., 1999), the Modified Fatigue Impact Scale (MFIS) (Fischer et al., 1999), and the Hospital Anxiety and Depression Scale (HADS) (Zigmond and Snaith, 1983). PDQ and MFIS are scales of the Multiple Sclerosis Quality of Life Inventory which measures patient perceptions of the so-called “MS invisible symptoms” and have good psychometric properties with demonstrated validity and reliability measures (Fischer et al., 1999). More specifically, PDQ and MFIS Cronbach's alpha (α) are reported to be 0.82 and 0.95, respectively (Ritvo et al., 1997). The PDQ is intended to measure cognitive deficits perceived by the patient by asking them about daily performance on the cognitive domains, most frequently affected in pwMS: attention–concentration, memory, and planning-organisation. The MFIS is a modified version of the Fatigue Impact Scale, which provides information about the impact of fatigue on three functioning domains: physical, cognitive, and psychosocial. The HADS is widely used in clinical practise and research for screening and monitoring anxiety and depression in a wide range of diseases, including MS. Consistency and test–retest measures for pwMS have been reported to be good (α between 0.82 and 0.85 for anxiety and depression sub-scale, respectively, and test–retest reliability intraclass correlation 0.83 for both sub-scales). Sensitivity and specificity range between 0.82 and 0.86, depending on the sub-scale and cutoff used (Marrie et al., 2018).

The Brief Repeatable Battery of Neuropsychological Tests (BRB-N) was used to objectively assess cognitive performance. It is a widely used battery specifically designed to be brief and target the most vulnerable cognitive functions in MS, widely used in clinical practise and research to cognitively assess pwMS with good psychometric properties (sensibility: 67–71%; specificity 85–94%) (Rao et al., 1990), and recognised by expert panels (Amato et al., 2018; Kalb et al., 2018; Higueras et al., 2022). It consists of the following subtests: Selective Reminding Test (SRT) that measures Long Term Storage (SRT LTS), Consistent Long Term Retrieval (SRT CLTR), and delayed recall (SRT R); the Spatial Reminding Test, total (SpaRT T) and delayed recall (SpaRT R), the Symbol Digit Modalities Test (SDMT), the Paced Auditory Serial Addition Test (PASAT), and the Word List Generation test (WLG). All assessments were performed by trained neuropsychologists. Clinical and demographical data (sex, age, educational level, MS phenotype, and disability—using the Expanded Disability Status Scale; EDSS and disease duration) were also collected. Raw scores of neuropsychological tests and PDQ were transformed into z scores, using HC mean and standard deviation. A global cognitive z score was created for each patient using the z scores obtained on the subtests of the Brief Repeatable Battery of Neuropsychological Tests, as previously described by Sepulcre et al. (2006).

As shown in Table 1A, participants were classified into three objective impairment groups according to their objective cognitive performance: (1) mild objective CI, if the global cognitive z score was between−1.0 and−1.5; (2) severe objective CI, if the z score was <-1.5; and (3) objectively preserved for any other z scores. Three subjective impairment groups were also created according to the subjective estimation of cognitive performance: (1) mild subjective CI, if the PDQ z score was between 1.0 and 1.5; (2) severe subjective CI, if the PDQ z score was >1.5; and (3) subjectively preserved for any other z scores.

**Table 1:** Objective and subjective impairment and accuracy categorisations.

**A. Objective and subjective cognitive performance**			
	**z score**		**Impairment**
Objective impairment (global cognitive z score)	−1.0 to−1.5	Mild CI	✓ Yes
	<-1.5	Severe CI	
	>-1.0	Preserved	✗ No
Subjective impairment (PDQ z score)	1.0 to 1.5	Mild CI	✓ Yes
	> 1.5	Severe CI	
	<1.0	Preserved	✗ No
**B. Transformation into accuracy**		
**Objective impairment**	**Subjective impairment**	**Accuracy**
✓ Yes	✓ Yes	Accurate estimator
✗ No	✗ No	
✗ No	✓ Yes	Underestimator
✓ Yes	✗ No	Overestimator

A: objective and subjective impairment classification, according to results on objective and subjective cognitive performance; B: accuracy classification from objective and subjective impairment into accuracy, according to consistency between objective and subjective evaluations.

To explore the accuracy of the subjective estimation of cognitive performance, patients were classified according to the degree of agreement they showed between objective and subjective measures. This was operationalised as follows (Table 1B): (1) accurate estimators, if they were both objectively and subjectively preserved or impaired, (2) underestimators, if they were objectively preserved but subjectively impaired; and (3) overestimators, if they were objectively impaired but subjectively preserved.

### Statistical analysis

Descriptive analysis and between-group comparison: The *t*-test or one-way analysis of variance, as appropriate for continuous variables, and the chi-square test, as appropriate for categorical variables, were performed for demographic and clinical data as well as for objective impairment, subjective impairment, and accuracy variables. Bonferroni corrections were performed in *post-hoc* tests. Categorical variables are expressed as frequencies and percentages. Continuous variables are expressed as mean ± standard deviation.

Linear regression models were used to compare cognitive performance between groups, adjusting results by age, educational level, and anxiety–depression, which are potential confounding variables since they are known to have a significant effect on cognitive performance. A linear regression model was performed for each dependent variable (raw score of the cognitive test). Variables included in each model were group (HCs, RRMS, or PMS), age, education years, and HADS score. Subjective perception and fatigue were also compared between groups using the same methodology.

To determine factors affecting the accuracy of subjective estimation among pwMS, univariate and bivariate analyses were performed using multinomial logistic regression. Accuracy was the dependent variable, and clinical and demographical data (sex, age, education years, MS phenotype, EDSS and disease duration, HADS, MFIS, and global cognitive z score) were included in the model as predictive variables. Variables known for having an effect on cognition and its perception were also included in the final model to permit adjustment of the results by these variables.

Significance was set at a *p*-value of < 0.05 for all statistical tests performed. IBM software SPSS^®^ Statistics v.23 was used to perform statistical analysis. Missing data were not taken into account for the analysis. No imputation was performed.

## Results

### Clinical and demographic characteristics across the groups

Clinical and demographical data are shown in Table 2. Significant differences were found regarding age and educational level. Both HC and RRMS patients were younger (42.56 ± 11.53 and 43.95 ± 9.76 years) than PMS patients (53.51 ± 7.59 years, *p* < 0.001). HC showed higher education years compared with RRMS and PMS patients (15.09 ± 3.01, 12.66 ± 3.43, and 11.11 ± 3.14 years, respectively, *p* < 0.001). Furthermore, the difference in education years between RRMS and PMS patients was also significant (*p* < 0.001). With respect to clinical data, higher EDSS in PMS compared with RRMS patients was observed (4.89 ± 1.43 vs. 2.03 ± 1.02, *p* < 0.001).

**Table 2:** Clinical and demographical data by group.

	**HC (*n* = 54)**	**RRMS (*n* = 65)**	**PMS (*n* = 47)**	***p***		
	**M (SD) /** ***n*** **(%)**	**M (SD) /** ***n*** **(%)**	**M (SD) /** ***n*** **(%)**	**PMS vs. HC**	**RRMS vs. HC**	**RRMS vs. PMS**
Male	22 (40.74)	21 (32.30)	19 (40.42)	0.574^a^		
Female	32 (59.26)	44 (67.70)	28 (59.58)
Age	42.56 ± 11.53	43.95 ± 9.76	53.51 ± 7.59	< 0.001^b^	1.000^b^	< 0.001^b^
Education years	15.09 ± 3.01	12.66 ± 3.43	11.11 ± 3.14	< 0.001^b^	< 0.001^b^	< 0.001^b^
Disease duration	-	9.52 ± 6.71	7.28 ± 7.30	-	-	0.095^c^
EDSS	-	2.03 ± 1.02	4.89 ± 1.43	-	-	< 0.001^c^

Categorical variables are presented by number and percentage. Continuous variables are presented by mean ± standard deviation ^*a*^Chi-square test; ^*b*^ANOVA post-hoc comparisons with Bonferroni correction; ^*c*^t-test. EDSS and disease duration variables do not apply to HC since they are disease-related variables. M, mean; SD, standard deviation; HC, healthy controls; RRMS, relapsing–remitting clinical course; PMS, progressive clinical course; EDSS, Expanded Disability Status Scale.

### Cognitive performance, perceived deficits, and fatigue across the groups

Table 3 shows the comparison of cognitive performance, fatigue, and perceived deficit between the groups. These differences were observed regardless of age, education level, and anxiety–depression scores. RRMS patients performed significantly worse than HCs on learning ability, information processing speed, and working memory. More concretely, on the SRT LTS (β = −7.05; *p* = 0.015), SDMT (β = −9.15; *p* = < 0,001), and PASAT (β = −4.43; *p* = 0.039), no significant differences were observed in perceived cognitive deficit measured with PDQ. Furthermore, RRMS patients had higher scores on MFIS than HCs (β = 9.76; *p* = 0.002). PMS patients performed significantly worse than HCs on verbal learning ability as well as verbal and visual memory (delayed recall), information processing speed, and working memory, more specifically, on the SRT LTS (β = −8.18; *p* = 0.016), SRT CLTR (β = −10.77; *p* = 0.002), SRT R (β = −1.76; *p* = 0.006), SpaRT R (β = −1.18; *p* = 0.027), SDMT (β = −14.22, *p* = < 0.001), and PASAT (β = −6.63; *p* = 0.010). Regarding perceived deficit, no differences were found between PMS patients and HCs. Higher MFIS scores were reported (β = 18.02; *p* = < 0.001) by the PMS group than HCs.

**Table 3:** Cognitive performance between-group comparisons. Results adjusted by age, education and anxiety-depression.

	**RRMS vs. HC**			**PMS vs. HC**		
	β**-coefficient**	**(95% CI)**	***p***	β**-coefficient**	**(95% CI)**	***p***
SRT LTS	−3.08	(-8.60, 2.43)	0.271	−8.18	(-14.82,−1.54)	0.016
SRT CLTR	−7.05	(-12.68,−1.41)	0.015	−10.77	(-17.55,−3.98)	0.002
SRT R	−0.85	(-1.88, 0.180)	0.105	−1.76	(-3.00,−0.52)	0.006
SpaRT T	−0.31	(-2.25, 1.62)	0.749	−1.69	(-4.02, 0.63)	0.153
SpaRT R	−0.29	(-1.15, 0.58)	0.514	−1.18	(-2.22,−0.14)	0.027
SDMT	−9.15	(-13.44,−4.86)	< 0.001	−14.22	(-19.39,−9.06)	< 0.001
PASAT	−4.43	(-8.61,−0.24)	0.039	−6.63	(-11.68,−1.59)	0.010
WLG	−0.41	(-2.60, 1.79)	0.716	−1.45	(-4.09, 1.19)	0.280
MFIS	9.76	(3.55, 15.98)	0.002	18.02	(10.54, 25.50)	< 0.001
PDQ	3.23	(-0.95, 7.40)	0.129	1.66	(-3.36, 6.69)	0.514

Single linear regression of each test raw score. Only the results of the factor group are shown. HC was the reference group. The results are adjusted by age, education, and the Hospital Anxiety and Depression Scale score. RRMS, relapsing–remitting clinical course; PMS, progressive clinical course; HC, healthy controls; CI, confidence interval; SRT LTS, Selective Reminding Test Long Term Storage; SRT CLTR, Selective Reminding Test Consistent Long Term Retrieval; SRT R, Selective Reminding Test Recall; SpaRT T, Spatial Reminding Test Total; SpaRT R, Spatial Reminding Test Recall; SDMT, Symbol Digit Modalities Test; PASAT, Paced Auditory Serial Addition Test; WLG, Word List Generation; MFIS, Modified Fatigue Impact Scale; PDQ, Perceived Deficit Questionnaire.

**Figure 1:**
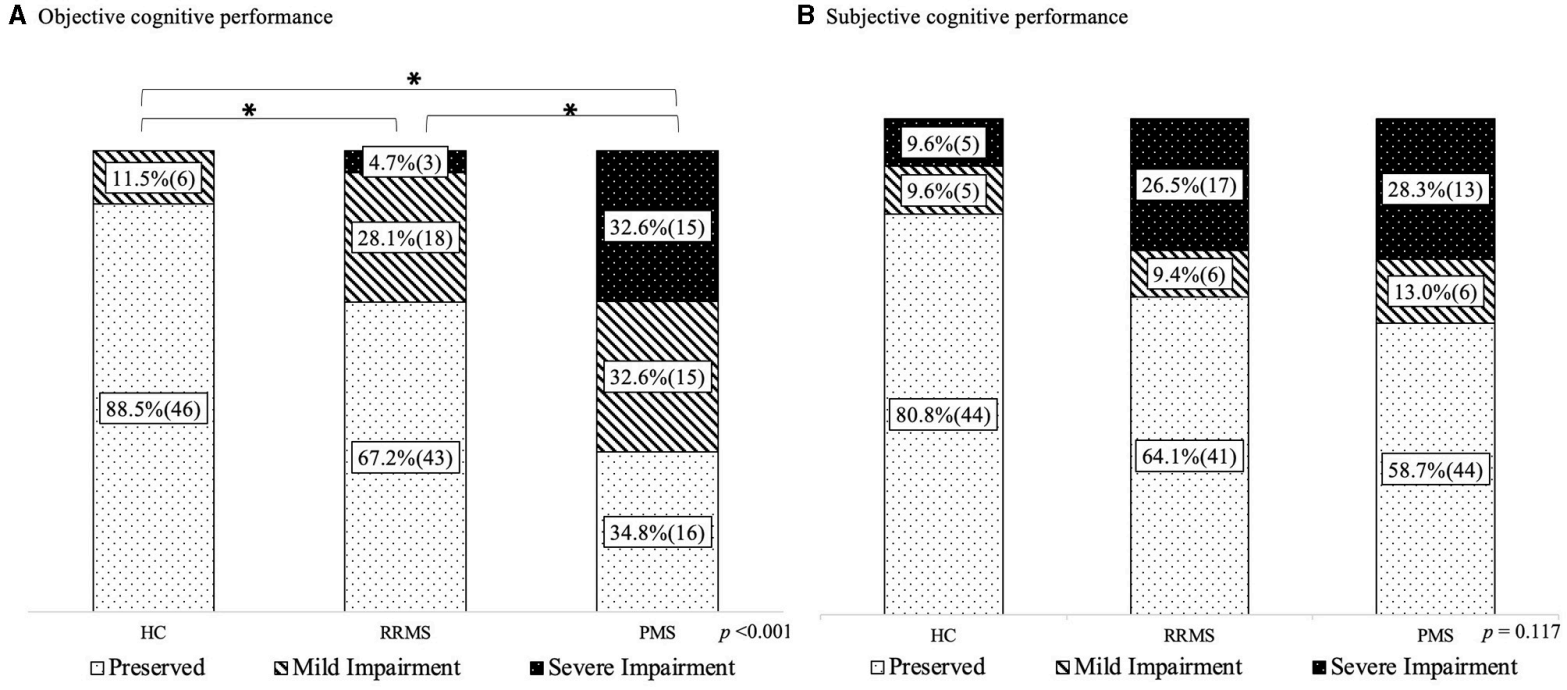
Objective and subjective impairment frequencies. **(A)** Objective cognitive performance. **(B)** Subjective cognitive performance. HC, healthy controls; RRMS, relapsing-remitting clinical course; PMS, progressive clinical course. % and (*n*) is presented; ^a^chi square test; *significant differences between groups in the reference category (objective preserved and subjective preserved, respectively).

Figure 1 shows the proportion of objective impairment (Figure 1A) and subjective impairment (Figure 1B) across the study groups. Both PMS and RRMS groups had more objective impairment than HCs (65.2 vs. 32.8% of RRMS and 11.5% of HC, *p* < 0.001). The PMS group also had a higher proportion of objectively impaired patients than the RRMS group. More specifically, the PMS group had a greater proportion of severe impairment than the RRMS (32.6 vs. 4.7%), but this difference was not found in the case of mild impairment (28.1% for RRMS vs. 32.6% for PMS). Conversely, there were no differences in the proportion of subjectively impaired patients among the HC, RRMS, and PMS groups.

### Accuracy in estimating cognitive abilities

**Figure 2:**
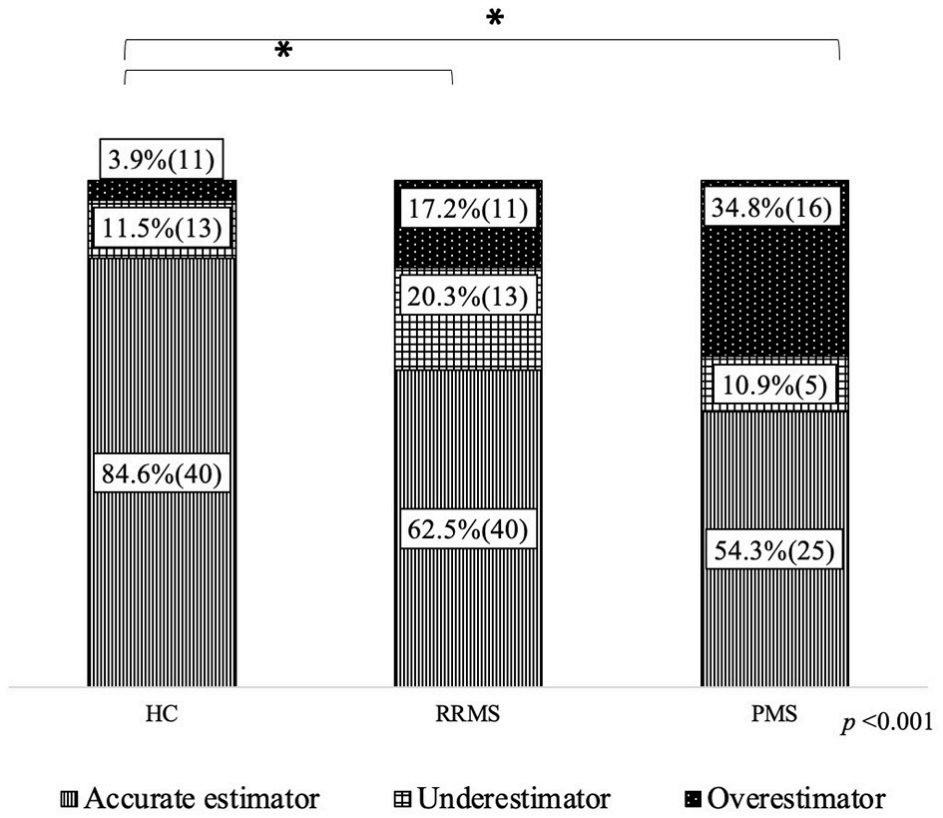
Accuracy in estimating cognitive performance. HC, healthy controls; RRMS, relapsing-remitting clinical course; PMS, progressive clinical course. % and (*n*) is presented; ^a^chi square test; *significant differences between groups in the reference category (accurate estimator).

Significant differences were found regarding accuracy (Figure 2). Both RRMS and PMS groups had a lower proportion of accurate estimators than HCs. The PMS group had higher ratios of overestimators (34.8%) than HCs (3.9%) and RRMS (17.2%), although the difference between the PMS and RRMS groups was not significant. There were no differences between the groups regarding underestimation.

Adjusted multinomial regression analysis (Table 4) showed MFIS (OR = 1.12; *p* < 0.001) and global cognitive z score (OR = 7.02; *p* = 0.003) to be risk factors for pwMS to become underestimators. Moreover, the EDSS score (OR = 1.53; *p* = 0.028) was found to be a risk factor for PwMS to become overestimators, while age (OR = 0.92; *p* = 0.024) and global cognitive z score (OR = 0.19; *p* = 0.001) were protective factors. Both protective and risk factors were found to be independent of age and education as well as all the other variables included in the model.

**Table 4:** Multinomial regression model of cognitive estimation accuracy. Univariate and bivariate analyses.

	**Accurate (*n* = 65; reference group)**	**Underestimators (*****n*** = **18)**					**Overestimators (*****n*** = **27)**				
			**Crude analysis**		**Adjusted analysis**			**Crude analysis**		**Adjusted analysis**	
	**M (SD)/*****n*** **(%)**	**M (SD)/*****n*** **(%)**	**OR (95%CI)**	***p***	**OR (95%CI)**	***p***	**M (SD)/*****n*** **(%)**	**OR (95%CI)**	***p***	**OR (95%CI)**	***p***
Age	48.12 (10.47)	47.33 (9.36)	0.99 (0.94, 1.05)	0.768	1.00 (0.93, 1.09)	0.863	48.59 (9.91)	1.01 (0.96, 1.05)	0.837	0.92 (0.86, 0.99)	0.024
Male	23 (35.4%)	5 (27.8%)	0.70 (0.22, 2.22)	0.547	-	-	12 (44.4%)	1.46 (0.59, 3.64)	0.416	-	-
Female	42 (64.6%)	13 (72.2%)	-		-	-	15 (55.6%)	-		-	-
Education years	12.28 (3.49)	11.94 (3.04)	0.97 (0.83, 1.14)	0.706	0.89 (0.70, 1.13)	0.346	11.26 (2.99)	0.91 (0.79, 1.05)	0.181	0.98 (0.82, 1.18)	0.837
Disease duration	11.28 (8.80)	13.44 (9.91)	1.03 (0.97, 1.09)	0.330	1.05 (0.97, 1.14)	0.237	12.88 (6.62)	1.02 (0.97, 1.08)	0.398	1.03 (0.96, 1.10)	0.425
EDSS	2.91 (1.82)	3.33 (1.45)	1.14 (0.85, 1.51)	0.381	0.88 (0.54, 1.44)	0.609	4.02 (2.05)	1.38 (1.08, 1.77)	0.011	1.53 (1.05, 2.23)	0.028
RRMS	40 (61.5%)	13 (72.2%)	1.63 (0.52, 5.11)	0.406	-	-	11 (40.7%)	0.43 (0.17, 1.07)	0.071	-	-
PMS	25 (38.5%)	5 (27.8%)	-		-	-	16 (59.3%)	-		-	-
HADS	10.68 (7.24)	13.83 (7.74)	1.06 (0.99, 1.14)	0.099	0.97 (0.87, 1.07)	0.504	10.30 (5.68)	0.99 (0.93, 1.06)	0.806	0.92 (0.82, 1.02)	0.108
MFIS	37.22 (21.72)	58.17 (11.63)	1.07 (1.03, 1.11)	0.001	1.12 (1.05, 1.19)	< 0.001	43.26 (20.29)	1.02 (0.99, 1.04)	0.206	0.99 (0.95, 1.02)	0.466
Cognitive z score	−0.76 (0.87)	−0.43 (0.46)	1.85 (0.89, 3.82)	0.099	7.02 (1.90, 25.94)	0.003	−1.41 (0.39)	0.27 (0.13, 0.57)	0.001	0.18 (0.06, 0.51)	0.001

M, mean; SD, standard deviation; OR, odds ratio; CI, confidence interval; EDSS, Expanded Disability Status Scale; RRMS, relapsing–remitting clinical course; PMS, progressive clinical course; HADS, Hospital Anxiety Depression Scale; MFIS, Modified Fatigue Impact Scale; cognitive z score: mean of z scores of all neuropsychological tests administered.

## Discussion

In this study, we have investigated perceived cognitive deficit across MS phenotypes in relationship with objective cognitive performance.

### Cognitive performance and metacognitive knowledge accuracy

We found that both RRMS and PMS patients performed significantly worse than HCs on cognitive tests, and cognitive impairment was more frequent in the RRMS and PMS groups than in HCs. Moreover, the PMS group performed worse than RRMS and had severe impairment more frequently. These findings are in line with current knowledge (Ruano et al., 2017; De Meo et al., 2021). However, the prevalence of CI in our study may have been slightly overestimated, since 11% of the HCs were categorised as cognitively impaired. Given the lack of consensus on how to define CI, we established mild impairment at more than 1.0 SD below the mean of HCs. This decision was made in order to avoid underestimation, mainly because the z score used was the mean of all cognitive tests, which would already blur mild or test-isolated low performances. In addition, significant differences in age and education between groups in our cohort could add some effect to this classification. However, these classifications were not made to study CI prevalence but rather the ulterior classification of accuracy, allowing us to study perceived cognitive performance, in terms of discrepancy in the SD from the mean of both objective and subjective measures. Thus, despite the fact that our data regarding the prevalence of CI should be interpreted with caution, the main results of our study, discussed below, are not affected.

Considering the proportion of perceived (subjective) cognitive impairment and the PDQ scores, no differences were found between the groups. Thus, these results, taken together, indicate that although cognitive disturbances are present in both RRMS and PMS groups, they might not be accurately perceived as they do not translate into more subjective complaints. This is also observed in the analysis of accuracy in the estimation of cognitive abilities, which is significantly less frequent in PMS and RRMS groups than in HCs. In addition, overestimation of cognitive abilities is much more frequent in the PMS group than HCs, whereas in the case of RRMS, there is no significant difference either with PMS or HCs. Indeed, most of the previous studies using a similar methodology (analysing accuracy, using either patient-informant or objective–subjective measures) did not take into account the MS phenotype, despite some of them including patients with different phenotypes. This fact, as well as the higher proportion of PMS patients in our sample and methodological differences regarding the categorisation of CI and accuracy, might explain differences in the accuracy, underestimation, and overestimation frequencies observed, which are higher in our sample than in other studies (Carone et al., 2005; Kinsinger et al., 2010; van der Hiele et al., 2012). However, a similar proportion of overestimation (~24%) was found in a 2010 study by Smith and Arnett. Sherman et al. (2008), who did take into account the MS phenotype although they used a different methodology, reported a similar prevalence of unawareness (31%) and also greater levels of unawareness in PMS than in RRMS. Rosti-Otajärvi et al. (2014) also included MS phenotype in the analysis, obtaining similar results regarding both absolute and group frequencies. Up to this point, our results suggest, in line with previous evidence (Mazancieux et al., 2019) despite methodological differences, that there are metacognitive knowledge impairments in pwMS both in the form of the underestimation and overestimation of cognitive abilities.

### Factors explaining metacognitive knowledge impairments

With regard to factors explaining impaired metacognitive knowledge, we found fatigue and cognitive performance to be risk factors for pwMS to become underestimators. Thus, as both fatigue and cognitive performance increase, the chances of perceiving cognitive performance that is lower than that which is objectively measured are also increased. For overestimation, which could be considered as unawareness or anosognosia, we found that EDSS is a risk factor. On the other hand, age and objective cognitive performance were protective factors. Therefore, regardless of the MS phenotype, the lower the cognitive performance, the greater the risk of becoming an overestimator. Interestingly, the MS phenotype alone was not powerful enough to explain inaccuracy through overestimation or underestimation. Hence, we believe that more accumulated disability (EDSS) and a higher prevalence of cognitive impairment may account for the augmented frequency of overestimators observed in PMS.

The cognitive demand of daily life activities could explain or contribute to the role of EDSS as a risk factor for overestimation, since patients with higher physical disability are probably less active in daily life activities, such as employment, caregiving, and housework, and less engaged in social activities. This may distort their perception of their cognitive abilities since they are confronted with their performance less often.

Our findings suggest that objective cognitive performance may play a critical role in metacognitive knowledge. This matches with the previous reports, indicating poorer awareness in the most cognitively impaired patients (Carone et al., 2005; Goverover et al., 2005; Sherman et al., 2008; Goverover et al., 2014; Rosti-Otajärvi et al., 2014) and theoretical models, together with intuition (Sherman et al., 2008; Mazancieux et al., 2019), which mainly propose that cognitive preservation is necessary to accurately monitor cognitive abilities. Accordingly, not only cognitive impairment might lead to anosognosia or lack of awareness of cognitive deficits but also cognitive preservation, as well as fatigue, may lead to cognitive subjective complaints that may not be otherwise objectively observed through neuropsychological evaluation.

We would like to highlight that our study suggests that fatigue plays a key role in the underestimation of cognitive performance. The available evidence about fatigue in metacognitive knowledge is scarce, and its role is still not fully understood, but there is data on its influence on subjective evaluations of one's own performance (Kinsinger et al., 2010; Jougleux-Vie et al., 2014; McNicholas et al., 2021). It is worth bearing in mind that fatigue may have a huge impact on daily activities, mainly through its impact on motivation, which cannot be reproduced in a less ecological situation such as neuropsychological assessment, besides the intrinsic differences in the cognitive demands between real-life tasks and cognitive evaluations. We suggest that the act of undergoing neuropsychological assessment may maximise motivation and effort, adding to discrepancies between the subjective perception of cognitive abilities, distorted by fatigue, and objective cognitive assessment.

Surprisingly, the anxiety–depression score was not significant in explaining inaccuracy in perceived cognitive deficit in our study. Although depression has been strongly correlated with perceived deficits in previous reports (Lovera et al., 2006; Julian et al., 2007; Kinsinger et al., 2010; Hanssen et al., 2014; Strober et al., 2016; Henneghan et al., 2017; McNicholas et al., 2021), as well as with inaccuracy in estimating cognitive performance (Carone et al., 2005), few studies have taken fatigue into account. Those that do include fatigue in their analyses find that metacognitive knowledge has a relationship with both depression and fatigue (Kinsinger et al., 2010; McNicholas et al., 2021). Many items on depression scales may be answered positively by pwMS suffering from fatigue since symptoms of depression and fatigue overlap (e.g., energy loss, slowness, and concentration difficulties) and, as mentioned, fatigue may have an impact on motivation as well as on activity engagement or enrolment (e.g., I still enjoy the things I used to enjoy). Thus, correlations reported between depression and perceived cognitive performance could disappear or attenuate when controlling for fatigue, as in our study.

### Clinical implications

Metacognition and cognition should both be considered, particularly in the current context of patient empowerment and involvement in disease management, given that it could be affected, especially by overestimation or unawareness of cognitive impairments. Metacognitive preservation is highly relevant in a chronic neurological condition, affecting young people with high cognitive demanding daily activities (active professional careers, socially engaged, family responsibilities, etc.). Deficit awareness is a critical factor in accurately monitoring cognitive performance in daily life and proactively seeking cognitive evaluations and treatments if needed. Equally important is active engagement in recommended treatments and rehabilitation strategies to maximise their effectiveness. Given that metacognitive impairments seem to be linked to cognitive impairments, it should be recommended to cognitively assess patients even if they do not have subjective complaints. Especially those with higher disability, which also seems to be related to deficit unawareness.

On the other hand, underestimation of cognitive abilities could lead to mood disturbances such as anxiety and quality of life deterioration. Beyond previous studies indicating a link among fatigue, cognitive performance, and subjective perceptions as well as reporting that cognitive rehabilitation therapies improve fatigue symptoms (Goverover et al., 2017; Grzegorski and Losy, 2017; Benedict et al., 2020); our results [suggesting an important role of fatigue in the underestimation of cognitive performance, in line with the study by Kinsinger et al. (2010)] imply that the treatment of fatigue may improve the accuracy of the estimation of cognitive performance. Thus, treatments with proven efficacy in improving fatigue such as cognitive behavioural therapy (van den Akker et al., 2016; Zarotti et al., 2022), physiotherapy, and exercise (Harrison et al., 2021) may also improve the accuracy on cognitive performance perceptions, lowering the risk of underestimation and its presumable impact on mood and quality of life. Additionally, more accurate metacognitive knowledge would lead to better disease management, given the improved ability of the patient to correctly monitor their cognitive performance and daily life activity functioning, helping detect incipient or subtle cognitive difficulties (before their severity contributes to the appearance of unawareness) that may not be detected by healthcare professionals and that could indicate disease activity or progression. Furthermore, it might have implications for cognitive rehabilitation therapy outcomes, given that the sooner it is applied, the better the outcomes.

### Limitations and future lines

Our study has some limitations. First of all, our study is single-centred, so biases inherent to the context or characteristics of the studied population may influence the results. Additionally, some limitations arise when studying perceived cognitive abilities in the performance of daily life activities compared with objective performance assessed in situations with low ecological validity, where there are factors, such as anxiety or motivation, that can potentially affect the performance. Moreover, our study neither has measures of other potential key explanatory variables such as brain reserve or cognitive demands on daily life activities nor neuroimaging parameters that usually present individual or between disease phenotype differences. Therefore, more studies with larger cohorts addressing these limitations are necessary not only to replicate these results but also to better understand this phenomenon, which has huge implications on aspects of disease management such as patient engagement and therapeutic adherence and patient quality of life. Ideally, studies would have a longitudinal design in order to take into account premorbid abilities in which patients may base their beliefs and opinions on how well they perform in cognitive abilities. This may be especially important for the study of underestimation phenomena, given that premorbid abilities may explain, at least in part, cognitive complaints that are not found in cognitive evaluations. An example of this might be where a patient's perceived cognitive performance is lower than at a previous evaluation but is found to be normal in comparison with reference populations. This can be seen usually in cases in which premorbid abilities are high (Sumowski et al., 2018). Future studies should also further investigate the role of fatigue, which has little evidence at present, and the specific cognitive domains involved in metacognitive knowledge and its underlying brain mechanisms. Bearing in mind the possibility that not all cognitive domains are equally involved in metacognition, it would also be of great interest to study the agreement between subjective cognitive complaints and objective cognitive performance, stratified by cognitive functions. Finally, possible differences in perceived deficits across different cognitive domains should also be explored.

## Conclusion

PwMS seem to have metacognitive knowledge impairments that lead to either anosognosia or subjective complaints that are not found by objective evaluation. This study provides new information about factors that explain these impairments as well as data on the prevalence in our cohort of under- and overestimation of cognitive abilities. Our results highlight that (1) anosognosia, which seems to be related to cognitive impairment, may be present in pwMS, regardless of the MS phenotype, and (2) fatigue appears to be a key factor in patients underestimating their cognitive abilities in our cohort of pwMS. Future studies are needed to better understand metacognitive impairments (underestimation and overestimation of cognitive abilities) in pwMS.

## Data availability statement

The raw data supporting the conclusions of this article will be made available by the authors, without undue reservation.

## Ethics statement

The studies involving human participants were reviewed and approved by CEIm Girona (Ethics Committee of Research with Medicines). The patients/participants provided their written informed consent to participate in this study.

## Author contributions

LR-T, JG, and RR-C conceived and designed the ConnectiMS project and study protocol. LR-T, RR-C, JG, CC-M, and JS-P recruited participants and collected the data. JG, CC-M, and JS-P conceived and designed this sub-study, with substantial contributions of all authors. CC-M, MB, EQ, and AQ-V planned and performed the statistical analysis. All authors discussed the results and contributed to the final manuscript.
