# Correction: Differences in metacognition between multiple sclerosis phenotypes: cognitive impairment and fatigue are key factors

**DOI:** 10.3389/fpsyg.2026.1837764

**Published:** 2026-04-13

**Authors:** Clàudia Coll-Martinez, Judit Salavedra-Pont, Maria Buxó, Ester Quintana, Ana Quiroga-Varela, René Robles-Cedeño, Marc Puig, Gary Álvarez-Bravo, Lluís Ramió-Torrentà, Jordi Gich

**Affiliations:** 1 Girona Neuroimmumology and Multiple Sclerosis Unit, Neurology Department, Dr. Josep Trueta University Hospital and Santa Caterina Hospital, Girona, Spain; 2 Neurodegeneration and Neuroinflammation Research Group, Girona Biomedical Research Institute (IDIBGI), Salt, Spain; 3 Redes de Investigación Cooperativa Orientada a Resultados en Salud (RICORS), Red de Enfermedades Inflamatorias (RD21/0002/0063), Instituto de Salud Carlos III, Madrid, Spain; 4 Statistical Unit, Girona Biomedical Research Institute (IDIBGI), Salt, Spain; 5 Medical Sciences Department, University of Girona, Girona, Spain

**Keywords:** cognition, metacognitive knowledge, deficit awareness, cognitive impairment, multiple sclerosis

In the published article, there was a mistake in the **Funding** statement. The funding statement was displayed as:

“This work was supported by the Departament de Salut of the Generalitat de Catalunya [SLT017/20/000115, 2021]; Instituto de Salud Carlos III, RICORS -Red de Enfermedades Inflamatorias [RD21/0002/0063, 2021].”

The correct Funding statement should read:

“This work was supported by the Departament de Salut of the Generalitat de Catalunya [SLT017/20/000115, 2021]; Instituto de Salud Carlos III through the project [RD21/0002/0063]; and co-funded by the European Union/Recovery, Transformation and Resilience Plan.”

The original version of this article has been updated.

